# The British Pharmacopœia, 1932
*Constable & Co., Ltd. Price 21s. 31s. with wide margins.


**Published:** 1932

**Authors:** A. L. Taylor

**Affiliations:** Pharmacist, Royal Infirmary, Bristol


### THE BRITISH PHARMACOPŒIA, 1932.*

BY

A. L. Taylor, M.P.S.,

*Pharmacist, Royal Infirmary, Bristol.*

---

The publication of a new *British Pharmacopœia* and its adoption as the official formulary always means some trouble for medical men and pharmacists alike. The difficulties are likely to be intensified in the case of this, the sixth edition, for two reasons: first, the fact that the book is not available for use until the end of September, although it becomes official on the 1st October; secondly, in that it embodies many more changes in form, size, and matter than in the case of any preceding edition—*e.g.* 358 articles are omitted that were in the 1914 B.P. and 128 included that were not. In 113 cases the name of the article has been altered from that of the 1914 B.P. In thirty-six cases the composition of the article has been altered from its predecessor, and in thirteen the strength of the article has been altered.

The omissions will perhaps arouse the greatest surprise, and at first sight possible dissatisfaction, when it is realized that the following old favourites of the prescriber no longer appear in the B.P. :—

Acetanilide.
Acid. Nitrohydrochlor. dil.
Aq. Laurocerasi.
Benzamine Lact.
Bismuth. Subnit.
Butyl Chloral Hyd.
Caffein. Citrat.
Decoct. Aloes Co.
Ext. Grindel. Liq.
Ext. Hydrast. Liq.
Hyd. Oxid. Rub.
Inf. Rosae Acid.
Inj. Morphin. Hypoderm.
Inj. Strychninae.
Inj. Apomorphinae.
Inj. Ergotae.
Liq. Bismuth et Ammon. Cit.

* Constable & Co., Ltd. Price 21s. 31s. with wide margins.

Mist. Cretæ.
Mist. Ferri Co.
Pil. Coloc. Co.
Pil. Ipecac. *c* Scill.
Pil. Saponis Co.
Pulv. Kino Co.
Salol.
Spirit. Armorac. Co.
Tinct. Cannab. Ind.
Tinct. Chlorof. et Morph. Co.
Tinct. Gelsemii.
Tinct. Opii Ammon.
Troch. Pot. Chlorat.
Ung. Gall. *c* Opio.
Vin. Ipecac. etc., etc.

The prescriber will doubtless be relieved to discover that, by mutual arrangement with the Committee of the "British Pharmaceutical Codex," the new edition of this work now approaching completion will contain most of these discards from the B.P.

The changes in the new book appear to be largely based on principle ; *e.g. all the vina or wines* have gone, in accordance with the international agreement "that no potent drug shall be prepared in the form of a medicinal wine." This has, of course, meant the scrapping of *vinum ipecacuanhæ*, which, however, reappears under the name of *tinct. ipecacuanhæ*.

All the old hypodermic injections have gone, doubtless on the assumption that the prescriber will prefer to use the more convenient hypodermic tablet of the particular strength needed.

A very large number of drugs, extracts, liquors, spirits and tinctures have been deleted, and in this connection a paragraph on page xx of the Introduction may be quoted as indicating the principles actuating the Pharmacopœia Commission, the compilers of the B.P. "In selecting the drugs to be included in the Pharmacopœia, the Commission has been guided by a desire to include only those substances which are of sufficient medicinal value to justify the continuance of their use by prescribers. *The mere fact that a drug is frequently prescribed has not been considered a sufficient justification for its inclusion.*" The italics are the reviewer's.

If, however, the list of omissions is long and remarkable, the additions to the 1932 B.P. are equally notable, as representing a desire to make the official formulary representative of modern ideas of medication and treatment. Among the more important may be noticed :—

Acid. Trichloracetic.
Acriflavine.
Agar-Agar.
Amidopyrin.
Amylocaine.
Cataplasma Kaolin.
Chloramine.
Chlorbutol.
Ephedrine Hydrochloride.
Ergotoxine Ethanesulphonate.
Erythrilis Tetanitras Dil.
Ext. Colch. Liq.
Ext. Hepat. Liq.
Ext. Hepat. Sicc. } The making of these two extracts official will fix a very necessary standard, and will tend to discourage the prescribing of a large number of preparations under fancy or proprietary names of unknown composition.
Ext. Pituitarii Liq.
Ext. Malt.
Ext. Malt. *c* Morrhuo.
Gelatin Zinc (Unna's Paste).
Ichthammol (Ichthyol).
Indicarmine (Indigo Carmine).
Infusa Recentia (*fresh* infusions).
Infusa Concentrata.

Concentrated infusions will always be used, it is to be feared, by doctor and chemist alike, and if so it is desirable to regularize their composition in the B.P.—*vide* page 9. In this connection it should be emphasized that if a prescriber wishes to have a fresh infusion used in his mixtures he *must* order "Inf. — *recent.*" If he does not do this the new official concentrated infusion may be used. This is an excellent innovation.

Insulin.
Liq. Ammon. Acet. Fort.
Liq. Ergosterol. Irrad.—Representing the first official recognition of the present enthusiasm for vitamine medication.
Liq. Iodi Fort.
Liq. Iodi Mitis. } Replacing the tinctures of the 1914 B.P.
Liq. Iodi Simplex.—Corresponding very closely with the old French Codex preparation, which, curiously enough, has been omitted from the new French Codex.
Neoarsphenamina.—The first of the organic arsenic preparations in the B.P.
Syr. Ferri. Phosph. Co.—" Parrish's."
Theophyllin Sod. Acet.

Official recognition of the use of gas is seen by the inclusion of :—

Aethylene,
Carbon Dioxide,
Nitrogen Monoxide,
Oxygen,
Aethyl Chloride, and of serum therapy by that of :—

Diphtheria } Antitoxins.
Tetanus. } Antitoxins.
Gas Gangrene. } Antitoxins.
(Schick Test Toxin). Toxin Diphther. Diagnostic.
Schick Control Toxin Diphther. Calefact.
Toxin Diphther. Detoxicat.
Tuberculins.
Vaccines.—T.A.B.
Vaccines.—Lymph.

The changes in form and substance of the monographs dealing with drugs and preparations represent, perhaps, the greatest advance in the B.P. The arrangements of sub-headings under characters :—

Tests for identity,
Tests for purity,
Assay,
Storage,
Sterilization, bring the book well up to the most modern standards; *vide* pages 8–11.

*Biological assays* of the following are included :—

(1) Antirachitic Vitamin D.
(2) Anti-dysentery Serum (Shiga).
(3) Diphtheria Antitoxin.
(4) Gas Gangrene Antitoxin.
(5) Tetanus Antitoxin.
(6) Insulin.
(7) Old Tuberculin.
(8) Pituitary (posterior) Ext.
(9) Digitalis.
(10) Strophanthin.
(11) Neoarsphenamine.
(12) Sulpharsphenamine.

Directions as to storage and sterilization are particularly desirable innovations.

The Appendices, twenty-one in number, may be regarded as a great advance on the 1914 B.P. in that they deal with :—

*Determination of pH values*.—Appendix iii includes a very useful and well-arranged table of indicators and their colour changes.

*Antimony trichloride* test for cod liver oil (Appendix xiv).

Special processes used in preparing solutions for injection (Appendix xvi) with especial reference to methods of sterilizing and tests for limit of alkalinity of glass.

Tests for freedom from living gas-producing organisms, anaerobic organisms, and undue toxicity.

The exclusive use of the metric system of weights and measures for formulae has been retained and doses are still given in metric and English systems. In this connection the amended doses of

Tinct. Belladonnæ.

Tinct. Digitalis.

Tinct. Nuc. Vom.

should be noted.

Certain changes in nomenclature must be noted, especially as in some cases there are also changes in composition :—

| B.P. 1914. | *becomes* | B.P. 1932. |
|---|---|---|
| Aether. Purif. | | Aether. Anaesthet. |
| Emp. Resini. | | Emp. Colophonii. |
| Liq. Trinitrini. | | Liq. Trinitratis. |
| Pulv. Ipecac. Co. (Dover's Powder). | | Pulv. Ipecac. et Opii. |
| Tinct. Camph. Co. | | Tinct. Camphorata. |
| Tinct. Iodi Fort. | | Liq. Iodi Fort. |
| Tinct. Iodi Mit. | | Liq. Iodi Mit. |

It is, perhaps, to be regretted that the date of the new B.P. coming into official use (1st October, 1932) should coincide so closely with the date on which it becomes available for purchase, but doubtless a wise latitude by prescribers and the authorities responsible for the administration of the Food and Drugs Act will be exercised, so that the transition from B.P. 1914 to B.P. 1932 may be made without undue difficulty.

